# Protein Translocation Acquires Substrate Selectivity Through ER Stress-Induced Reassembly of Translocon Auxiliary Components

**DOI:** 10.3390/cells9020518

**Published:** 2020-02-24

**Authors:** Sohee Lee, Yejin Shin, Kyunggon Kim, Youngsup Song, Yongsub Kim, Sang-Wook Kang

**Affiliations:** 1 Department of Biomedical Sciences, University of Ulsan College of Medicine, Seoul 138-736, Korea; vshw3358@naver.com (S.L.); uo127@naver.com (Y.S.); ysong@amc.seoul.kr (Y.S.);; 2 Asan Medical Institute of Convergence Science and Technology, Asan Medical Center, Seoul 05505, Korea; 3 Department of Convergence Medicine, Asan Medical Center, Seoul 05505, Korea; kimkyunggon@gmail.com

**Keywords:** protein translocation, protein quality control, prion protein, ER stress, redox homeostasis

## Abstract

Protein import across the endoplasmic reticulum membrane is physiologically regulated in a substrate-selective manner to ensure the protection of stressed ER from the overload of misfolded proteins. However, it is poorly understood how different types of substrates are accurately distinguished and disqualified during translocational regulation. In this study, we found poorly assembled translocon-associated protein (TRAP) complexes in stressed ER. Immunoaffinity purification identified calnexin in the TRAP complex in which poor assembly inhibited membrane insertion of the prion protein (PrP) in a transmembrane sequence-selective manner, through translocational regulation. This reaction was induced selectively by redox perturbation, rather than calcium depletion, in the ER. The liberation of ERp57 from calnexin appeared to be the reason for the redox sensitivity. Stress-independent disruption of the TRAP complex prevented a pathogenic transmembrane form of PrP (ctmPrP) from accumulating in the ER. This study uncovered a previously unappreciated role for calnexin in assisting the redox-sensitive function of the TRAP complex and provided insights into the ER stress-induced reassembly of translocon auxiliary components as a key mechanism by which protein translocation acquires substrate selectivity.

## 1. Introduction 

The majority of secreted and membrane proteins synthesized in mammalian cells are co-translationally transported across or inserted into the endoplasmic reticulum (ER) membrane (termed, “translocation”) [1]. This event occurs at a specialized domain (termed, “translocon”), which serves as a gateway for nascent polypeptides entering the ER. It is densely assembled with hetero-trimeric protein-conducting Sec61 channels (SecYEG in eubacteria, SecYEβ in archaea) and nearby translocon auxiliary components (TACs) [2,3]. 

Protein translocation involves two sequential steps, protein targeting and gating. At the targeting step, the ribosome-nascent chain complex is recognized by the signal recognition particle (SRP), thereby pausing translation, and is delivered to the ER membrane via the specific interaction of SRP with the SRP receptor (SR). At the gating step, the nascent polypeptide liberated from the SRP resumes translation and enters the ER through the central pore of the Sec61 complex [4]. Between these two steps, protein translocation can be regulated at the gating step and its efficiency appears to be determined by the sequence fidelity of the hydrophobic signal sequence and/or the internal transmembrane domain (TMD) in the nascent polypeptides entering the ER (hereafter, referred to as “substrates”) [4,5]. 

We have previously demonstrated that this gating step is a physiologically regulated process in response to ER stress. During ER stress, nascent prion protein (PrP), bearing an inherently inefficient signal sequence, is released from the Sec61 complex and into the cytosol for proteasomal degradation. This regulation is advantageous for cells in that it is a pathway for pre-emptive quality control (pQC), which reduces the burden of misfolded PrP entering the stressed ER [6]. In our recent study, a similar regulatory mechanism was also found to be used for the selective degradation of PrP with a pathogenic TMD mutation (ctmPrP) [7]. 

In addition to these protein-specific sequence elements, protein translocation can also be regulated by the architecture and dynamics of the Sec61 complex. It has been well described conceptually and experimentally that the transient conformational change of the Sec61 complex can be mediated by dynamic interactions with TACs, such as the OST complex, TRAM, the TRAP complex, RAMP4, and the Sec62/63 complex, which are often altered by different types of substrates [8,9]. Indeed, the TRAP complex facilitates the initiation of PrP translocation [10]. In a similar context, the PrP signal sequence stabilizes the interaction of Sec61 with Sec62 and Sec63, but the prolactin (Prl) signal sequence (i.e., an inherently efficient signal sequence) does not [11]. 

More recently, a new TAC has been identified. IRE1α, a membrane-integrated ER stress sensor with RNase activity, forms a complex with Sec61 and efficiently binds and cleaves *XBP1* mRNA recruited to the Sec61 complex through its nascent chain [12]. An unprecedented role of Sec62 has also been discovered. Sec62 serves as a autophagy receptor, delivering misfolded ER proteins to the autophagy pathway and contributing to restoring the ER from the results of stress conditions [13]. Given that both TACs play unique roles in maintaining ER homeostasis, we hypothesized that there are cell type-specific connections between ER stress and TAC assemblies and that this is the reason for different activities of the Sec61 complex. 

Intensive biochemical and structural analyses of the translocon in native ER membranes have demonstrated that the TRAP complex not only interacts directly with translocating nascent polypeptides, translating ribosomes, and protein foldases, but also often interacts with the Sec61 complex, depending on the signal sequence [11,14,15,16]. We, therefore, hypothesized that TRAPα may be a promising checkpoint to maintain protein homeostasis in the ER. Among the four TRAP subunits (i.e., α, β, γ, and δ subunit), TRAPα is the best characterized and is the main subunit of the TRAP complex. Silencing TRAPα reduces TRAPβ and γ subunit levels, destabilizes the TRAP complex, and often perturbs membrane protein topologenesis in a TMD sequence-sensitive manner at the ER membrane [17]. 

This study was motivated by the result showing the loss of PrP translocation regulation in TRAPα-deficient cells. This unanticipated result raises an important question for a previously unappreciated component in the TRAP complex. Here, we identified TRAPα-bound membrane proteins and aimed to determine their functional links to the outcomes of ER stress, using PrP as a reporter.

## 2. Materials and Methods

### 2.1. Antibodies and Reagents

The following antibodies were used in this study: anti-Sec61α (1:5000), anti-Sec61β (1:5000), anti-TRAPα (1:5000), anti-SRα (1:5000), anti-FLAG (1:1000), and anti-PrP-A antibodies (1:5000), which have been previously described [7,10,18]. Anti-clanexin (1:1000), anti-BiP (1:1000), and anti-calreticulin antibodies (1:1000) were obtained from Cell Signaling Technology (Danvers, MA, USA). Anti-PDI (1:5000) and anti-HA-conjugated magnetic beads were obtained from Thermo Fisher Scientific (Waltham, MA, USA). A PrP-specific 3F4 antibody (1:10,000) was purchased from BioLegend (San Diego, CA, USA). Anti-FLAG (M2)-conjugated magnetic beads, FLAG peptide, DTT, thapsigargin (Tg), and all chemicals for biochemical analyses were purchased from Sigma-Aldrich Korea (Seoul, Korea). 

### 2.2. Molecular Biology

All mutant PrP constructs, including PrP-Prl, Prl–PrP, N7a-PrP, PrP(-SP), PrP-ASGR(-SP), N7a-PrP-AV3, N7a-PrP-ASGR, and their G34N mutants were created from hamster prion protein (PRNP) cDNA (GenBank accession no.: EF_139168; https://www.ncbi.nlm.nih.gov/genbank/), cloned in the pcDNA5/FRT/To vector (Thermo Fisher Scientific), by conventional site-directed mutagenesis using Phusion high-fidelity DNA polymerase (New England Biolabs, Ipswich, MA, USA) [4,5,7]. Mutant constructs of calnexin (NM_001024649) and ERp57 (NM_005313) were also engineered in the same manner. The various constructs of TRAPα (NM_003144), Sec61β (NM_006808), calnexin, and ERp57, fused with FLAG or HA, were generated by inserting their PCR-amplified cDNAs into HindIII/XhoI or EcoRV/XhoI sites of the homemade pcDNA5/FRT/TO-3xFLAG or HA vectors. All enzymes used for cloning were purchased from New England Biolabs. 

sgRNA constructs for CRISPR/Cas9 genome editing were created by the insertion of phosphorylated synthetic oligos targeting TRAPα (GPP sgRNA designer; https://portals. broadinstitute.org/gpp/public/analysis-tools/sgrna-design; sgTRAP1#1-S: 5′-CACCGGGTGGCACT ACAGTGTTCAG-3′, sgTRAP1#1-AS: 5′-AAACCTGAACACTGTAGTGCCACCC-3′, sgTRAP1#2-S: 5′-CACCGCCAAATGGTCGTCCGCCCAT-3′, sgTRAP1#2-AS: 5′-AAACATGGGCGGACGACCATT TGGC-3′, sgTRAP1#3-S: 5′-CACCGAGTATAGTTGTATCTGCACT-3′, sgTRAP1#3-AS: 5′-AAACAG TGCAGATACAACTATACTC-3′) into the BsmB1 site of the lentiGuide-puro vector, a gift from Feng Zhang (Addgene, Watertown, MA, USA; plasmid #52963). For endogenous calnexin mutation, an sgRNA construct was created in the same manner, but the synthetic oligos were designed to target the region near the sequence encoding cysteine at codon 160 of calnexin (sgCANX-C160-S: 5′-CACCGTTTAGAAAGCAGTTTCACAT-3′; sgCANX-C160-AS: 5′-AAACATGTGAAACTGCTTTCT AAAC-3′). T7 endonuclease 1 (T7E1)-based heteroduplex cleavage assays were performed according to the protocol provided by ToolGen (Seoul, Korea). All constructs were confirmed by DNA sequencing (Cosmogenetech, Seoul, Korea). 

### 2.3. Cell Culture Analyses

Flp-In T-REx 293 cells were purchased from Invitrogen (Carlsbad, CA, USA), grown in Dulbecco’s modified Eagle medium, supplemented with 10% fetal calf serum in 5% CO_2_ at 37 °C, and transfected with Lipofectamine 2000 (Invitrogen, Carlsbad, CA, USA). 

Isogenic Flp-In T-REx 293 cell lines inducibly expressing wild-type and mutant calnexin, and TRAPα fused with HA were generated according to the manufacturer’s instructions. In this system, the CMV promoter, controlled by PrP expression, was induced by doxycycline (10 ng/mL) for 16 h, unless otherwise indicated. To establish CRISPR/Cas9 cell lines, Flp-In T-REx 293 cells were co-transfected with lentiGuide-puro containing target gRNAs and pcDNA-Cas9-G418. Cell lines were established by the clonal isolation of antibiotic-resistant cells by serial dilution in 96 well plates. The gene edits were verified using T7E1 assay and immunoblotting. Wild-type or mutant PrPs were transiently transfected into these cell lines and induced by doxycycline (10 ng/mL) for 16 h, unless otherwise indicated. 

Colony-forming assays were performed using a previously published procedure, with minor modifications [19]. Briefly, cells (100 cells/well) were plated on 35 mm dishes and cultured for 3 weeks. Viable cell colonies were fixed, counter-stained with 6% glutaraldehyde containing 0.5% crystal violet, and visualized via GelCount^TM^ (Oxford Optronix; Abington, UK), using the manufacturer’s image acquisition software. 

### 2.4. Biochemistry

PrP synthesis and turnover rates were assessed by pulse-chase experiments in cells inducibly expressing wild-type and mutant PrPs. Cells were starved with serum-free and methionine/cysteine-free media for 15 min and pulse-labeled with a *trans*-labeling mixture ([^35^S]-methionine/cysteine; PerkinElmer, Waltham, MA, USA) for an additional 15 min, unless otherwise indicated. Following replacement of the media with normal growth media, pulse-labeled cells were harvested at the indicated time points. Cells were washed once with 1 × PBS, fully solubilized in buffer K (1% SDS, 100 mM Tris-HCl, pH 7.5) with boiling, and diluted 10× with IPT buffer (1% Triton X-100, 50 mM HEPES, pH 7.5, 150 mM NaCl). Diluted lysates were incubated for 90 min with PrP-specific 3F4 or PrP-A antibodies and then incubated with protein G-conjugated magnetic beads (GE Healthcare Life Sciences, Marlborough, MA, USA) for an additional 90 min at 4 °C. Beads were washed five times with 1 mL of IPT buffer and suspended in 30 µL of 1.5 × SDS-PAGE sample buffer. Samples (10 µL) were separated on gels and visualized using autoradiography. 

All procedures for protein isolation were performed in a cold chamber (4 °C) or on ice. Immunoaffinity purification was performed according to previously published procedures, with minor modifications [12]. Briefly, cells expressing TRAPα fused with HA in cell culture dishes (150 mm) were washed twice with 1× PBS, resuspended in buffer A (10 mM HEPES, pH 7.4; 250 mM sucrose; 2 mM MgCl_2_) containing 1× *C*omplete protease inhibitor cocktail (Roche, Basel, Switzerland), and homogenized by 60 repeated passages through a 23-gauge needle. Cell homogenates were centrifuged for 30 min at 3000× *g* to remove cell debris and the rough microsome fractions were collected from the supernatant by centrifugation for 1 h at 75,000× *g* in a TLA-100.3 rotor (Beckman Coulter, Brea, CA, USA). Microsomes were carefully resuspended in 100 µL of buffer B (10 mM HEPES, pH 7.4; 250 mM sucrose; 2 mM MgCl_2_; 0.5 mM DTT) and solubilized in lysis buffer (50 mM HEPES, pH 7.4; 5 mM MgAc; 1 mM DTT; 150 mM NaCl; 2% digitonin) for 30 min. After centrifugation for 15 min at 20,000× *g*, the supernatant was incubated with anti-HA-conjugated magnetic beads and stringently washed six times with lysis buffer containing 0.2% digitonin. Proteins bound to the beads were eluted using buffer E (0.1 M glycine, pH 2.3 and 0.5% Triton X-100). Eluents were neutralized in ten volumes of 1 M Tris-HCl, pH 7.5 and precipitated with TCA. For protein identification, eluents were separated by SDS-PAGE and stained with GelCode Blue Stain Reagent (Thermo Fisher Scientific). Differentially recovered bands were excised, then identified by mass spec analysis (ProteomeTech, Seoul, Korea). The individual spectra from MS/MS were processed using the SEQUEST software (Thermo Quest, San Jose, CA, USA) and the generated peak lists were used to query NCBI database using the MASCOT program (Matrix Science Ltd., London, UK).

Co-immunoprecipitation studies were performed in stable/inducible cell lines expressing wild-type or mutant calnexin fused with HA. Following transfection with TRAPα-FLAG or ERp57-FLAG constructs, cells were washed once with 1× PBS and solubilized in IPM buffer (2% digitonin; 50 mM HEPES, pH 7.5; 150 mM NaCl; 5 mM MgOAc; 1 mM DTT) containing 1× *C*omplete protease inhibitor cocktail. After centrifugation for 10 min at 6000× *g* to remove cell debris, detergent-soluble lysates were incubated for 3 h with anti-FLAG (M2)- or anti-HA-conjugated magnetic beads. Beads were stringently washed six times with 1 mL of IPM buffer and resuspended in 30 µL of 1.5× SDS-PAGE sample buffer. Ten microliters of sample were separated on gels and analyzed by immunoblotting with the indicated antibodies.

In experiments where total protein was being analyzed, cells at each time point were fully solubilized in buffer K, with boiling, and then processed further. The exact times and conditions of each experiment are described in individual figure legends.

## 3. Results

### 3.1. TRAPα Is Not the Key Element in PrP Synthesis

**Figure 1:**
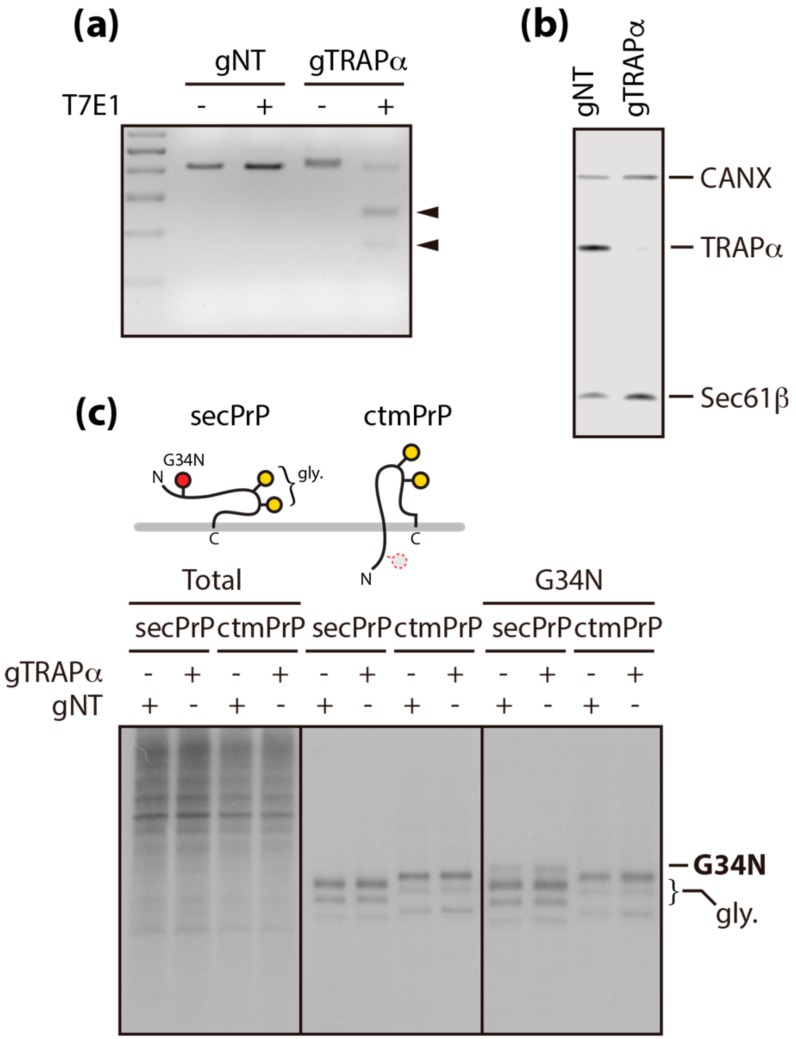
Analyses of newly synthesized PrP in TRAPα-deficient cells. (**a**) A TRAPα-deficient cell line (gTRAPα) was created using CRISPR/Cas9 genome editing, using a TRAPα-targeting sgRNA. Gene editing was confirmed using T7E1 assay. Heteroduplex fragments cleaved by T7E1 are indicated as arrowheads. gNT: cell line expressing non-targeting gRNA as a negative control. (**b**) Specific elimination of TRAPα protein was verified in fully solubilized TRAPα-deficient cells by immunoblotting with calnexin (CANX)-, Sec61β-, and TRAPα-specific antibodies. (**c**) Topological differences between secPrP and ctmPrP are illustrated in the upper panel. Newly synthesized secPrP and ctmPrP in pulse-labeled gNT and gTRAPα cells transiently transfected with wtPrP (secPrP) or N7a-PrP-AV3 (ctmPrP) constructs were analyzed by immunoprecipitation with the PrP-specific 3F4 antibody (lower middle panel). In this manner, the luminal localization of the N-terminal region was determined in these cell lines expressing mutant PrPs carrying the G34N mutation (lower right panel). Equal loading and translation were verified by determining total newly synthesized protein content in the cells (lower left panel).

To better understand the impact of TRAPα in protein translocation, we created a TRAPα-deficient cell line using CRISPR/Cas9 genome editing with a TRAPα-targeting sgRNA (hereafter, referred to as “gTRAPα”). As a negative control, we cloned an additional CRISPR/Cas9 cell line using a non-targeting sgRNA (hereafter, referred to as “gNT”). TRAPα gene editing and its selective deficiency were confirmed using a T7E1-based heteroduplex cleavage assay (Figure 1A) and by the absence of detectable TRAPα protein (Figure 1B), respectively. This cell line was used to determine whether TRAPα was required for the translocation of PrP. 

Four features make PrP a particularly useful model for this study. Firstly, because PrP is a typical GPI-anchored protein that is generally poorly degraded by the ER-associated degradation pathway, its translocation efficiency can be determined by simply monitoring its expression levels [20]. Secondly, in contrast to prolactin (Prl), PrP has an inherently inefficient signal sequence that is destabilized during ER stress [6]. Thirdly, PrP is a well-known substrate of the TRAP complex [10]. Finally, PrP has two N-linked glycan acceptor sites that are modified in the ER, thus facilitating the assessment of its translocation into the ER [21].

PrP is synthesized in three topogenic isoforms, corresponding to secPrP, ntmPrP, and ctmPrP. The synthesis of these isoforms is mainly determined by the fidelity of the signal sequence, but is often determined by a combinatorial control mechanism involving the internal hydrophobic region during translocation [5]. The present study focused on secPrP and ctmPrP, because their synthesis levels have previously been found to be regulated by translocation and topogenesis, respectively [6,7]. These isoforms were expressed in TRAPα-deficient cells and their translocation efficiencies were assessed by monitoring the levels of newly synthesized fully glycosylated forms, in pulse-labeled cells. However, unlike a previous in vitro membrane reconstitution study demonstrating that TRAP depletion destabilizes the PrP signal sequence and inhibits PrP translocation into the ER [10], newly synthesized secPrP levels were unchanged in TRAPα-deficient pulse-labeled cells. This lack of an effect on secPrP levels was also seen in the ctmPrP-favoring mutant and further verified by monitoring the modification of an N-linked glycosylation acceptor site (G34N) introduced into the N-terminal region of PrPs (Figure 1C). These results indicated that TRAPα may not be the key element regulating the translocation and membrane insertion of PrP in cultured cells. 

### 3.2. Calnexin is a Component of the TRAP Complex

This discrepancy between results derived from the cell-free system and cultured cells prompted us to hypothesize that an uncharacterized additional component exists in the TRAP complex to assist it in its function. Given that ER stress inhibits PrP translocation into the ER [6], we hypothesized that the TRAP complex may be disrupted by ER stress inducers. We tested this hypothesis by the comparative analysis of TRAPα-interacting membrane proteins in the presence and absence of ER stress. 

**Figure 2:**
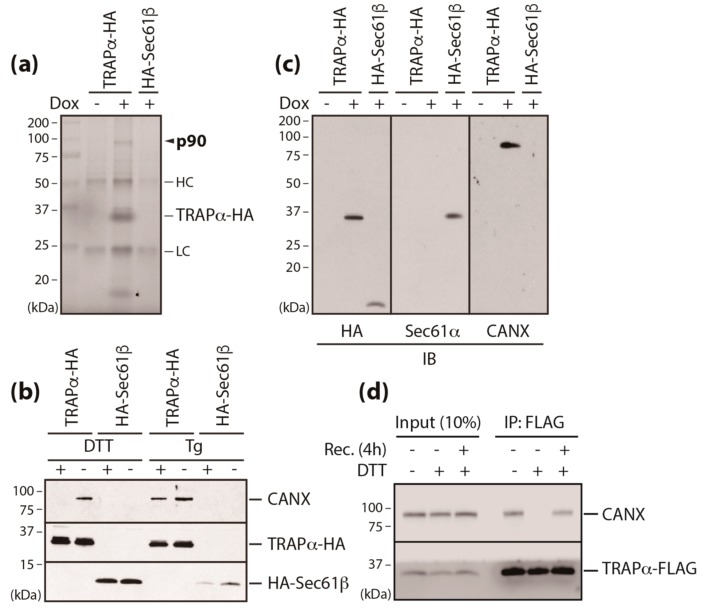
Redox-sensitive interaction of calnexin and translocon-associated protein (TRAP) α (**a**) TRAPα complexes were recovered with anti-HA magnetic beads from detergent-solubilized microsomes isolated from stable/inducible cells expressing TRAPα-HA after doxycycline treatment (Dox; 10 ng/mL). HC: immunoglobulin heavy chain, LC: immunoglobulin light chain. (**b**) Immunoaffinity purification was performed as in (**a**) in cells treated with DTT (10 mM) or thapsigargin (Tg; 5 µM) for 1 h or 4 h, respectively. Recovery of the indicated ER membrane proteins was determined by immunoblotting with anti-HA and anti-calnexin antibodies. (**c**) TRAPα complexes recovered in (**a**) were subjected to immunoblotting with anti-HA, anti-Sec61α, and anti-calnexin antibodies. (**d**) The interaction between calnexin and TRAPα was analyzed in cells transiently transfected with a TRAPα-FLAG construct. Cells treated with DTT (10 mM) for 1 h were allowed to recover for 4 h in the absence of DTT, before being solubilized in IPM buffer. The restored interaction was assessed by detecting calnexin in TRAPα-interacting molecules precipitated with anti-FLAG antibody-conjugated magnetic beads.

To this end, we employed immunoaffinity purification of detergent-solubilized microsomes isolated from isogenic Flp-In 293 T-Rex cell lines expressing TRAPα fused with HA (TRAPα-HA) and found that p90 was selectively recovered with TRAPα (Figure 2A, Figure S1). This interaction was disrupted by DTT, a redox reagent that prevents disulfide formation, but was not affected by Tg, which perturbs calcium homeostasis in the ER (Figure 2B). p90 was identified as calnexin (CANX), an ER-resident chaperone, by mass spectrophotometry and its identity was further confirmed by immunoblotting with a specific anti-CANX antibody (Figure 2B). However, CANX did not appear to be a core component of the translocon complex, as evidenced by its failure to co-immunoprecipitation with either Sec61α or Sec61β (Figure 2C). The perturbed interaction between CANX and TRAPα was recovered by the removal of DTT (Figure 2D), suggesting that the interaction is redox-sensitive. 

### 3.3. A Conserved Disulfide Bridge within CANX Provides the Interaction with TRAPα

**Figure 3:**
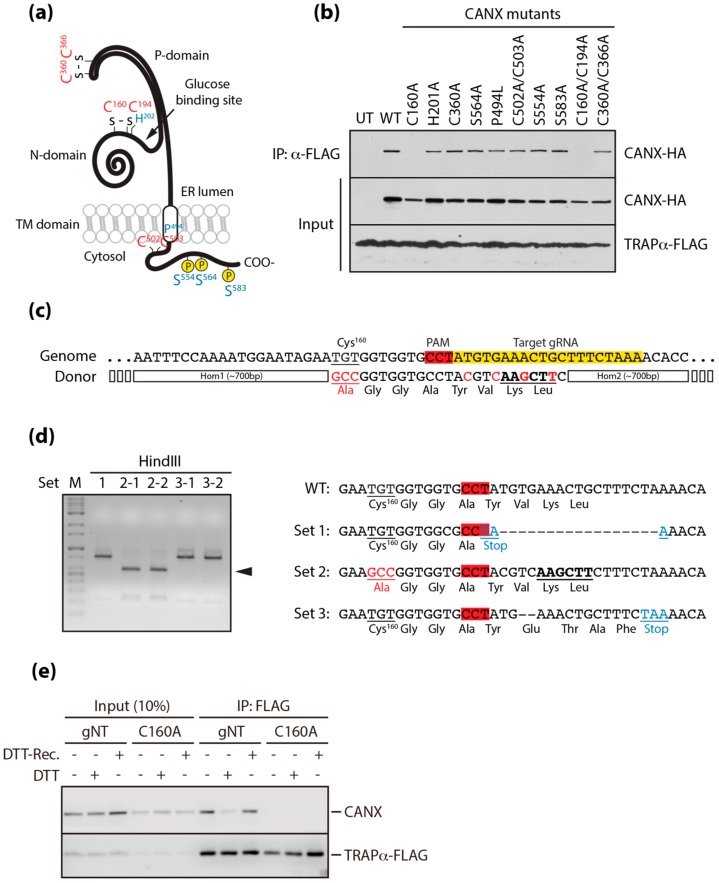
Development of stress-independent PATC. (**a**) Functional residues of the primary sequence of calnexin. (**b**) The residue in calnexin required for the interaction with TRAPα was determined by co-immunoprecipitation in cells expressing various calnexin mutants fused with HA, as described in Figure 2D. (**c**) The sgRNA targeting the region near the sequence encoding cysteine at codon 160 of calnexin (Yellow box). (**d**) Desired genome editing (C160A mutation) as in (**c**) was confirmed by the detection of the DNA fragment cleaved by HindIII (arrowhead, left panel) and DNA sequence analysis (right panel). (**e**) Stress-independent disruption of the interaction between calnexin and TRAPα in C160A cells was verified by co-immunoprecipitation, as described in Figure 2D.

There are multiple amino acid residues within the primary sequence of CANX that are functionally important for its different activities [22,23,24]. We created constructs carrying mutations of these residues, to determine the amino acid responsible for the interaction between CANX and TRAPα (Figure 3A). These mutants were fused with HA, expressed in cells expressing TRAPα fused with FLAG, and precipitated with an anti-FLAG antibody. All mutants were successfully recovered, except those carrying a highly conserved cysteine residue at codon 160 (Figure 3B), suggesting the need for a conformationally intact lectin domain cluster in the glucose binding pocket of CANX for the interaction with TRAPα [25,26]. 

Testing the direct impact of the disrupted CANX and TRAPα interaction requires excluding additional reactions caused by redox perturbation of the ER. To create a stress-independent disruption of the interaction, we constructed a cell line endogenously expressing mutant CANX (C160A), using CRISPR/Cas9-mediated gene editing with a specific guide RNA targeting the region near the sequence encoding cysteine at codon 160 and a donor plasmid carrying a cassette of the C160A mutation fused with homology arms. Furthermore, by creating silent mutations with the insertion of a HindIII site, we protected the target sequence carrying the C160A mutation in the donor plasmid from being targeted by the gRNA (Figure 3C). Gene editing was verified by a T7E1-based heteroduplex cleavage assay (data not shown), by detecting the fragment cleaved by Hind III, and by sequencing PCR-amplified genomic DNA near the target site (C160A) (Figure 3D). We eventually produced a cell line endogenously expressing mutant CANX (C160A) that did not interact with TRAPα, even under normal conditions or during the recovery from DTT-induced stress (Figure 3E). Nevertheless, it has been shown that mutating CANX does not affect the typical outcomes of ER stress (Figure S2A), the turnover rates of TRAPα and CANX (Figure S2B), or the subcellular localization of CANX (Figure S2C). Thus, this cell line bears ***p***oorly ***a***ssembled ***T***RAP ***c***omplexes (hereafter, referred to as “PATC”), independent of ER stress. 

### 3.4. PATC Interferes with ctmPrP Synthesis

CANX assists N-linked glycoproteins to fold properly and serves to keep aberrantly folded glycoproteins in the stressed ER, preventing further transport to the secretory pathway [27]. However, the turnover rate, metabolism, and processing of newly synthesized PrP in pulse-labeled cells were unchanged by the mutation of CANX (C160A), even under ER stress. Similar results were seen for clusterin (CLU), a highly glycosylated secretory protein, in pulse-chase experiments performed in the same manner (Figure S3). In these experiments, the native signal sequences of PrP and CLU were replaced with the Prl signal sequence to ensure efficient translocation, even after ER stress [6]. 

**Figure 4:**
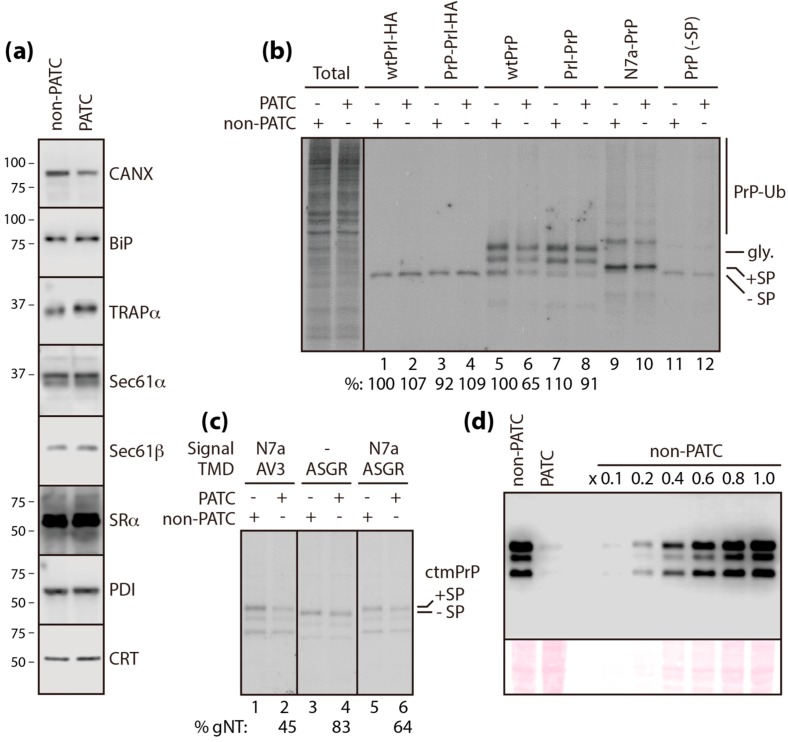
Analysis of PrP synthesis in PATC. (**a**) Fully solubilized non-PATC and PATC cells were subjected to immunoblotting with the indicated antibodies. (**b**) Prolactin fused with HA and newly synthesized PrP isoforms in pulse-labeled non-PATC and PATC cells transiently transfected with various mutant constructs were analyzed by immunoprecipitation with anti-HA and PrP-specific 3F4 antibodies. PrP-Ub: ubiquitinated subpopulation of cytPrP, gly: glycosylated subpopulation of PrP in the ER, +SP/-SP: uncleaved/cleaved signal sequence. (**c**) Newly synthesized ctmPrP in pulse-labeled non-PATC and PATC cells transiently transfected with the indicated constructs was analyzed by immunoprecipitation with an anti-PrP-A antibody. (**d**) The amount of ctmPrP accumulated in non-PATC and PATC cells was titrated by immunoblotting with the 3F4 antibody (upper panel). Serial dilution of protein loading was confirmed by staining the blot with Ponceaus S (lower panel).

Given that the TRAP complex facilitates the translocation of PrP into the ER [10], we hypothesized that CANX, identified as a component of the TRAP complex in the present study, may be functionally involved in PrP translocation. Firstly, the mutation of CANX did not affect the expression of the core translocon components and major ER chaperones tested (Figure 4A, Figure S4). Next, we assessed the translocation efficiency of PrP in cells with normal (i.e., wild-type CANX, hereafter, referred to as “non-PATC cells”) or poorly assembled TRAP complexes (i.e., mutant CANX [C160A], hereafter, referred to as “PATC cells”). PrP has two N-glycan acceptor sites that are co-translationally modified in the ER. Therefore, we compared the levels of newly synthesized glycosylated PrP in pulse-labeled non-PATC and PATC cells, to determine the relative efficiency of PrP translocation. In non-PATC cells, fully glycosylated PrP and Prl levels were unchanged, even after swapping their signal sequences (Figure 4B, lane 1–4). By contrast, the levels of newly synthesized fully glycosylated wild-type PrP were reduced by up to ~71% in PATC cells compared to non-PATC cells (lane 5 vs 6), but were restored by up to ~91% by replacing the native signal sequence of PrP with that of Prl (“Prl–PrP”, lane 5 vs 8). These results, observed selectively in PATC cells, suggested that stress-independent PATC (i.e., C160A mutation) had a similar effect to ER stress [6]. However, this regulation did not seem to depend solely on the signal sequence, because the levels of newly synthesized Prl in PATC cells were unchanged, despite replacing the native signal sequence of Prl with that of PrP (“PrP–Prl”, lane 2 vs 4). This result suggested the involvement of the mature domain in this regulation. 

In addition to secretory PrP synthesized from wtPrP and Prl–PrP, two additional topogenic isoforms corresponding to cytosolic (cytPrP) and transmembrane (ctmPrP) forms of PrP are often synthesized under conditions of stress and this topogenic heterogeneity is thought to be governed by translocational regulation [21,28]. Both isoforms can be experimentally produced by point mutations within the N-terminal signal sequence and/or the internal hydrophobic domain of PrP [5,7]. Comparative analyses of newly synthesized PrP in pulse-labeled non-PATC and PATC cells expressing cytPrP (“N7a-PrP” and “PrP lacking signal sequence”) revealed that cytPrP synthesis was insensitive to PATC mutation (Figure 4B, lane 9~12). By contrast, ctmPrP (“N7a-PrP-AV3”) synthesis was the most susceptible to PATC mutation among the various PrP isoforms (i.e., glycosylated secPrP vs ctmPrP = ~71% vs ~45%; Figure 4B, lane 6 vs Figure 4C, lane 2), resulting in an accumulation of only ~ 10% of ctmPrP in non-PATC cells (Figure 4D).

The ctmPrP-favoring mutant used in this study had a point mutation within the N-terminal signal sequence (“N7a”) and the internal hydrophobic domain (“AV3”), which serve as sequence motifs for ER targeting and membrane insertion, respectively [4,5]. Combinatorial control by these motifs, through translocational regulation, allows PrP to undergo topologic conversion and membrane insertion [5,7]. In this context, our observation that newly synthesized ctmPrP was reduced in PATC cells, suggested that PATC interfered with the membrane insertion of PrP mediated by the AV3 mutation. This notion seems to be plausible, because the AV3 mutation exhibits low fidelity in membrane insertion [7]. This was tested using a domain-swapping assay, in which the internal hydrophobic domain carrying the AV3 mutation of the ctmPrP-favoring mutant was replaced with a typical transmembrane domain (TMD) of the asialoglycoprotein receptor (ASGR) to ensure efficient membrane insertion. As evidenced by the restored levels of newly synthesized ctmPrP in pulsed-labeled PATC cells by the replacement of the TMD (Figure 4C, lane 2 vs lane 6), PATC inhibited the membrane insertion of ctmPrP in a TMD-selective manner. This reaction was not a mechanism for post-translational clearance of pathogenic ctmPrP accumulated in the ER, as evidenced by the similar turnover rates of newly synthesized ctmPrP in non-PATC and PATC cells (Figure S5). Given that the levels of ctmPrP (ASGR) lacking the signal sequence were restored in PATC cells (Figure 4C, lane 3 vs 4), PATC seems to interfere with the interaction between the signal sequence and the TMD during translocation. This may be a plausible explanation for the reduced ctmPrP levels in PATC cells. 

### 3.5. PATC Disrupts the Interaction of ERp57 with CANX

**Figure 5:**
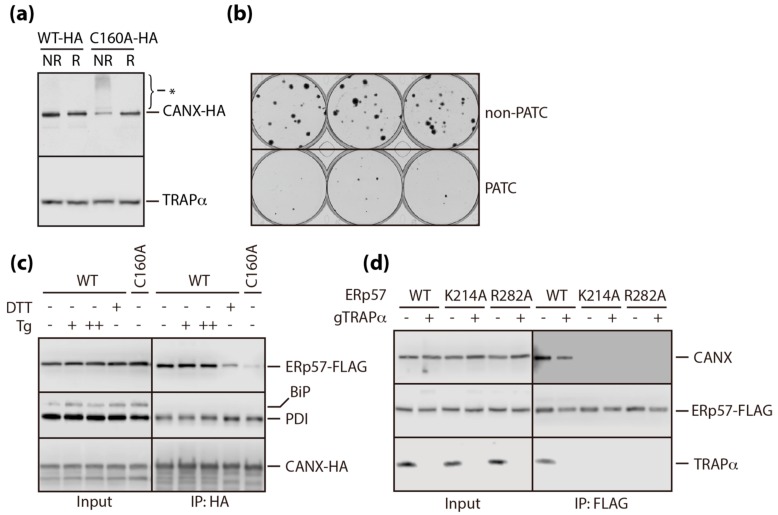
Analyses of the interaction between ERp57 and calnexin. (**a**) Fully solubilized stable/inducible cells expressing wild-type (WT) or mutant calnexin (C160A) fused with HA were subjected to immunoblotting with an anti-HA antibody, under reducing (R) and non-reducing (NR) conditions. Equal loading was confirmed by probing the same blot with an anti-TRAPα antibody. (**b**) gNT and C160A cell lines (100 cells/well) were plated in triplicate and visualized 3 weeks later by staining with crystal violet. (**c**) Stable/inducible cells expressing wild-type (WT) or mutant calnexin (C160A) fused with HA were transiently transfected with an ERp57-FLAG construct and treated with DTT (10 mM) for 1 h or thapsigargin (Tg, 1 µM =”+” or 5 µM = ”++”) for 4 h, before being solubilized in IPM buffer. The interactions of ERp57, BiP, and PDI with calnexin were determined by immunoblotting with specific antibodies in calnexin-interacting molecules precipitated with anti-HA antibody-conjugated magnetic beads. (**d**) gNT and gTRAPα cells were transiently transfected with mutant ERp57 (K214A, R282A)-FLAG constructs. The interactions of calnexin and TRAPα with wild-type or mutant ERp57 were determined by immunoblotting with specific antibodies in ERp57-interacting molecules precipitated with anti-FLAG antibody-conjugated magnetic beads.

Redox-sensitive dissociation of CANX from TRAPα appears to be the central mechanism underlying PATC. However, in contrast to CANX, there is no redox-active cysteine residue in the primary amino acid sequence of TRAPα. Therefore, a redox-sensitive conformational change of CANX may be the main the reason for PATC. This was confirmed by the detection of an additional subpopulation of SDS-resistant CANX oligomers in PATC cell lysates under non-reducing conditions (Figure 5A). Persistent accumulation of CANX (C160A) oligomers led to cytotoxicity, as measured by the number of colonies formed from viable cells (Figure 5B). 

**Figure 6:**
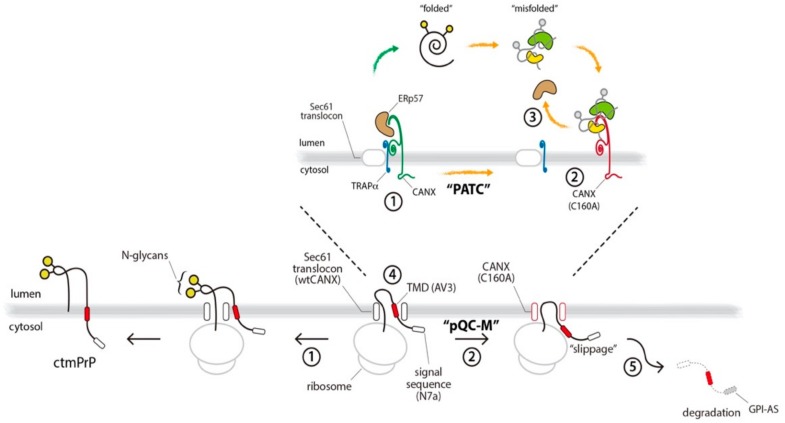
Working model depicting the functional link between PATC and pQC-M. Redox-sensitive selective inhibition of ctmPrP synthesis through translocational regulation is illustrated.

To identify the factor involved in the oligomerization of CANX (C160A), we focused on the redox-active ER chaperones, PDI and ERp57, and found that their interactions with CANX had differential sensitivities to redox perturbation. The interaction of PDI with CANX was not affected by ER stress inducers or CANX mutation. By contrast, the interaction of ERp57, the closest known homologue of PDI, with wild-type CANX was considerably weakened by redox perturbation (DTT), but not by the depletion of calcium (Tg) in the ER. Disruption of the interaction between CANX and ERp57 was more obvious in PATC cells, suggesting that this interaction is directly associated with redox-sensitive PATC (Figure 5C). This interaction was completely abrogated by mutation of two well-characterized functional residues, K214 and R282 [29,30], within ERp57, but was not affected in TRAPα-deficient cells (Figure 5D). These results provided an insight into the role of ERp57 as a key factor in the crosstalk between protein folding and translocation, which have previously been considered as two separate redox-sensitive responses compromised by ER stress (Figure 6).

## 4. Discussion

In this study, we showed that the reassembly of translocon auxiliary components induced by ER stress is the main mechanism by which protein translocation acquires substrate selectivity. Our intensive biochemical analyses identified a component of the TRAP complex and highlighted a previously unappreciated role of this component in the redox perturbation of the ER. The major findings of the present study are two-fold. Firstly, calnexin (CANX) directly interacted with TRAPα in the TRAP complex. Secondly, redox-sensitive disruption of this interaction in the TRAP complex inhibited the co-translational membrane insertion of ctmPrP. This regulation seemed to be a pathway for the pre-emptive quality control of membrane proteins (hereafter, referred to as “pQC-M” to distinguish it from the pQC of secretory proteins, in which translocations are regulated by signal sequence efficiency) and ensured the protection of the stressed ER from the overload of pathogenic ctmPrP, through translocational regulation (Figure 6). This study provided clues to help answer several unresolved questions on the substrate-specific regulation of protein translocation into the ER. 

Firstly, which steps in the pQC-M process are susceptible to redox perturbation? Here, we demonstrated that CANX formed a complex with TRAPα in the TRAP complex adjacent to the Sec61 channel. Because the CANX/TRAPα complex has been shown not to interact directly with the Sec61 channel under normal conditions (i.e., targeting), the interaction of the ribosome-nascent chain and Sec61 seems not to be perturbed by CANX. This may be an acceptable explanation for the unchanged ratio of glycosylated proteins to cytosolic proteins in PATC cells (Figure S6). When translation restarts and the nascent chain passes through the Sec61 channel (i.e, translocation), the translocating nascent polypeptide interacts directly with the TRAP complex, where the dynamic transition of the interaction between CANX and TRAPα seems to change the conformation of the TRAP complex, for ctmPrP translocation, to a permissive (Figure 6, step 1) or non-permissive state (step 2). In the present study, we found that ERp57 served as a trigger of this reaction. The mechanistic basis for this involves the coupling of ERp57 to CANX under normal conditions (step 1), but its release from CANX upon redox perturbation of the ER (step 3). Given the fact that ERp57 interacts with newly synthesized integral membrane proteins bearing N-glycans, in combination with CANX, the selective inhibition of ctmPrP synthesis may occur due to the redox-sensitive disruption of the interaction of ERp57 with CANX [31,32]. Thus, ERp57 is an additional redox-sensitive component of the TRAP complex, and the proper assembly of CANX-ERp57-TRAPα in the TRAP complex ensures the efficient membrane insertion of ctmPrP, through translocational regulation (Figure 6). 

Secondly, how is pQC-M regulated to influence the synthesis of ctmPrP? Our analyses allowed us to identify several important determinants that route ctmPrP to pQC-M, as follows. Firstly, PATC induces the pQC-M of ctmPrP (step 2). Considering our results in TRAPα-deficient cells, the efficient translocation of PrP appears to be due to the proper assembly of the TRAP complex, rather than the individual functions of the TRAPα subunit. Instead, we found that CANX interacted with TRAPα and we demonstrated that their interaction facilitated the membrane insertion of ctmPrP, through translocational regulation (step 1). Secondly, an unstable TMD within ctmPrP appears to be a sequence element that activates pQC-M. It is well-known that PrP bears an inherently less efficient N-terminal signal sequence that is destabilized in conditions of stress [6]. In contrast to secPrP, ctmPrP contains an additional sequence element, the internal hydrophobic region, which acts as a TMD and contributes to its unique topology [5,21]. Our results in PATC cells showing that ctmPrP synthesis was recovered by an increase in TMD fidelity (ASGR-TMD), suggested that pQC-M is regulated in a TMD-selective manner (step 2). Finally, pQC-M is regulated in response to redox perturbation of the ER. We found here that proper assembly of CANX in the TRAP complex required a cysteine thiol modification within CANX, because it was disrupted by the C160A mutation. pQC-M does not appear to be a common mechanism caused by all types of ER stress, but it appears to be specific to redox perturbation that can be recovered by the withdrawal of DTT. 

Finally, what is the importance of pQC-M to cells? Among the three topogenic isoforms of PrP, ctmPrP is the form that is the most pathogenic when it accumulates [33,34]. To prevent the accumulation of ctmPrP, cells activate multiple quality control pathways specialized for ctmPrP [5,7]. This study suggested that pQC-M is one such mechanism to maintain PrP homeostasis during ER stress. pQC-M recognizes unstable TMD and leads ctmPrP to undergo slippage from the Sec61 complex during translocation (step 4). This pathway is advantageous to cells in at least two aspects. Firstly, it ensures the folding capacity of the stressed ER is maintained, by reducing the burden of pathogenic ctmPrP entering the ER. Secondly, pQC-M results in the selective degradation of ctmPrP. pQC-M reroutes ctmPrP to the cytosol, where the fully translated ctmPrP, bearing hydrophobic sequence elements, an N-terminal signal sequence, an internal hydrophobic region, and a C-terminal GPI-anchored sequence, is rapidly degraded via the proteasome-dependent pathway (step 5). In this context, pQC-M ensures the protection of the ER from the accumulation of ctmPrP during conditions of stress. However, it is not known whether persistent pQC-M is beneficial for cells, based on the fact that stress-independent PATC results in the accumulation of CANX oligomers and leads to cytotoxicity (Figure 5B). 

Among the various questions that remain to be addressed, the structural basis of the redox-sensitive combinatorial interaction of CANX-ERp57-TRAPα and the functional link between these interactions and substrate-specific proteostasis control through translocational regulation, remain important questions for future studies.

## Supplementary Materials

The following are available online at https://www.mdpi.com/2073-4409/9/2/518/s1, Figure S1: Identification of p90 by LC-MS/MS; Figure S2: Effect of PATC on the typical outcomes of ER stress; Figure S3: Effect of PATC on PrP metabolism and processing; Figure S4: Effect of PATC on the expression of the core translocon components and major ER chaperones; Figure S5: Effect of PATC on ctmPrP turnover; Figure S6: Effect of PATC on DTT-induced translational repression.
