# Supplementary material for: Protein Translocation Acquires Substrate Selectivity Through ER Stress-Induced Reassembly of Translocon Auxiliary Components
Source: Cells. 2020 Feb 24;9(2):518. doi: 10.3390/cells9020518 (PMC7072789; doi:10.3390/cells9020518)
Supplement: Supplementary file 1 [file cells-09-00518-s001.pdf]

Supplementary Materials

Protein Translocation Acquires Substrate Selectivity through ER Stress-Induced Reassembly of Translocon Auxiliary Components

Sohee Lee, Yejin Shin, Kyunggon Kim, Youngsup Song, Yongsub Kim and Sang-Wook Kang

(a)

gi|179832	**calnexin [Homo sapiens]**
gi|28590	unnamed protein product [Homo sapiens]
gi|21361306	BDNF/NT-3 growth factors receptor isoform a precursor [Homo sapiens]
gi|7547029	GAP-like protein [Homo sapiens]]
gi|47458048	hemojuvelin isoform a precursor [Homo sapiens]

(b)

```
  1 MEGKWLLCML LVLGTAIVEA HDGHDDDVID IEDDLDDVIE EVEDSKPDTT APPSSPKVTY
 61 KAPVPTGEVY FADSFDRGTL SGWILSKAKK DDTDDEIAKY DGKWEVEEMK ESKLPGDKGL
121 VLMSRAKHHA ISAKLNKPFL FDTKPLIVQY EVNFQNGIEC GGAYVKLLSK TPELNLDQFH
181 DKTPYTIMFG PDKCGEDYKL HFIFRHKNPK TGIYEEKHAK RPDADLKTYF TDKKTHLYTL
241 ILNPDNSFEI LVDQSVVNSG NLLNDMTPPV NPSREIEDPE DRKPEDWDER PKIPDPEAVK
301 PDDWDEDAPA KIPDEEATKP EGWLDDEPEY VPDPDAEKPE DWDEDMDGEW EAPQIANPRC
361 ESAPGCGVWQ RPVIDNPNYK GKWKPPMIDN PSYQGIWKPR KIPNPDFFED LEPFRMTPFS
421 AIGLELWSMT SDIFFDNFII CADRRIVDDW ANDGWGLKKA ADGAAEPGVV GQMIEAAEER
481 PWLWVVYILT VALPVFLVIL FCCSGKKQTS GMEYKKTDAP QPDVKEEEEE KEEEKDKGDE
541 EEEGEEKLEE KQKSDAEEDG GTVSQEEEDR KPKAEEDEIL NRSPRNRKPR RE
```

(c)

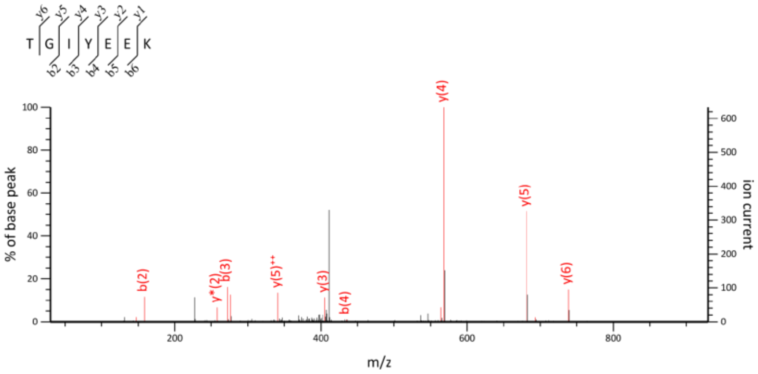

Figure S1. Identification of p90 by LC-MS/MS. (**a**) Significant hits as defined by MASCOT probability analysis were listed. (**b**) The primary sequence coverage of calnexin obtained by analysis of p90-derived peptides from mass spectrometry was illustrated. Sequenced peptides are shown in red. (**c**) MS/MS spectrum of a tryptic peptide, TGIYEEK, with the highest score (underline as in **b**) was presented.

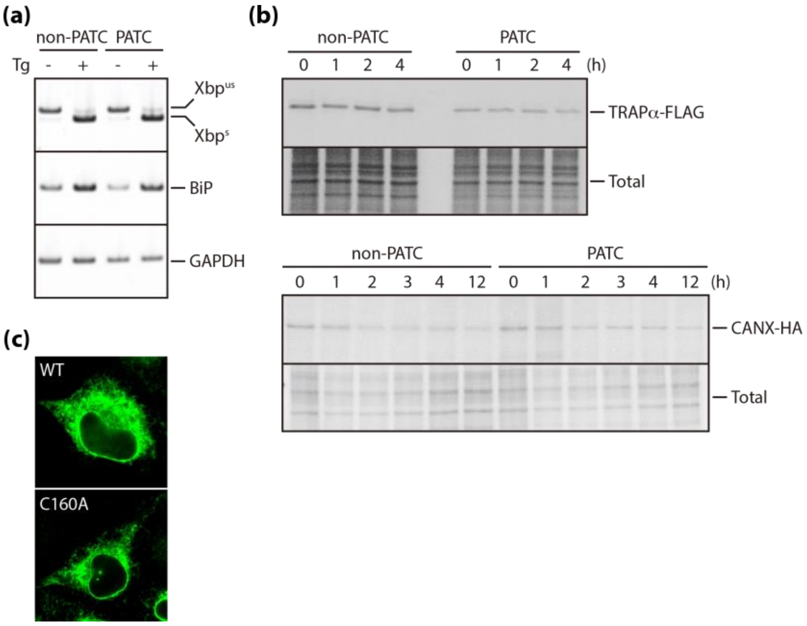

Figure S2. Effect of PATC on the typical outcomes of ER stress. (**a**) ER stress-induced UPR activation in non-PATC and PATC cells was verified by increased levels of spliced *XBP1* mRNA and *BiP* mRNA, as detected using RT-PCR. (**b**) The synthesis and turnover rates of newly synthesized TRAPα (upper panel) and calnexin (lower panel) were determined by pulse-chase experiments in non-PATC and PATC cells expressing TRAPα-FLAG and calnexin-HA. Fully solubilized cells harvested at the indicated time points during the chase were subjected to immunoprecipitation with anti-FLAG or anti-HA antibodies. (**c**) Subcellular distributions of wild-type (WT) and mutant calnexin (C160A) were visualized by the direct fluorescence of GFP in HeLa cells transiently transfected with GFP-fused calnexin constructs.

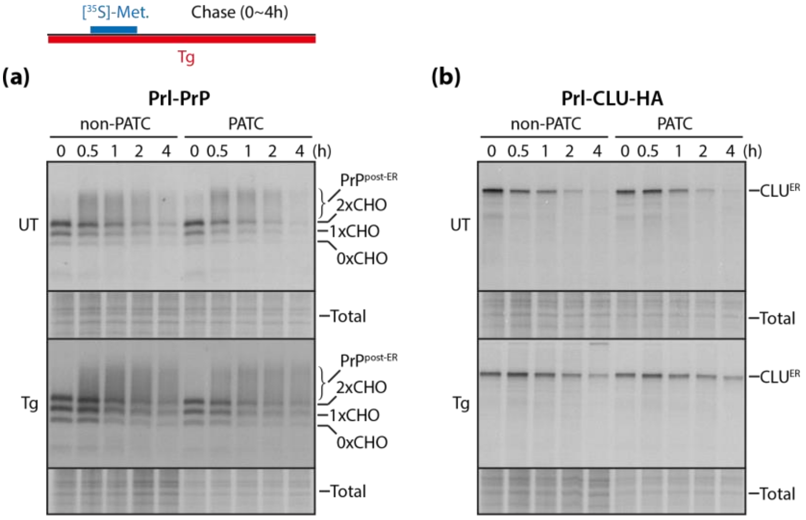

Figure S3. Effect of PATC on PrP metabolism and processing. (**a**) Post-translational metabolism, processing, and turnover of newly synthesized PrP were analyzed by pulse-chase experiments in non-PATC and PATC cells transiently transfected with Prl-PrP in the presence or absence of thapsigargin (5 μM), as illustrated. Fully solubilized cells harvested at the indicated time points during the chase were subjected to immunoprecipitation with the PrP-specific 3F4 antibody. (**b**) Clusterin fused with HA was also analyzed as in (**a**), but was recovered with an anti-HA antibody.

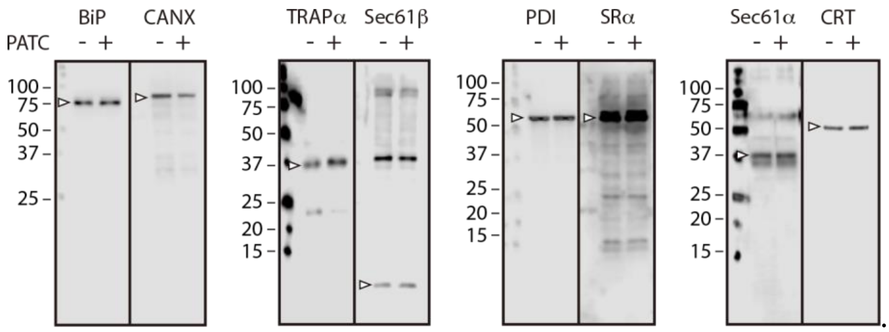

Figure S4. Effect of PATC on the expression of the core translocon components and major ER chaperones. Full blots of Fig 4A were presented.

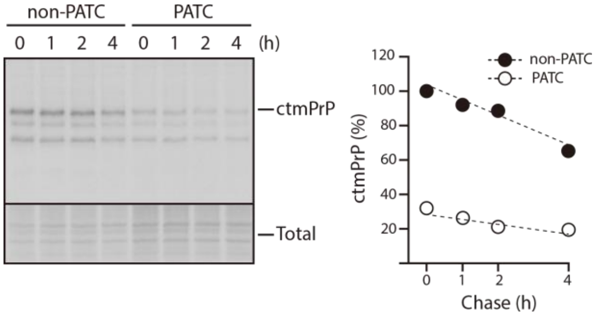

Figure S5. Effect of PATC on ctmPrP turnover. The synthesis and turnover rate of newly synthesized ctmPrP were determined by pulse-chase experiments in non-PATC and PATC cells stably expressing ctmPrP-favoring mutants. Fully solubilized cells harvested at the indicated time points during the chase were subjected to immunoprecipitation with the PrP-specific 3F4 antibody (left panel). The ctmPrP band densities on the gel were quantified using Image J software (National Institutes of Health, Bethesda, MD, USA) and expressed as a percentage of the amount of PrP labeled at pulse (right panel).

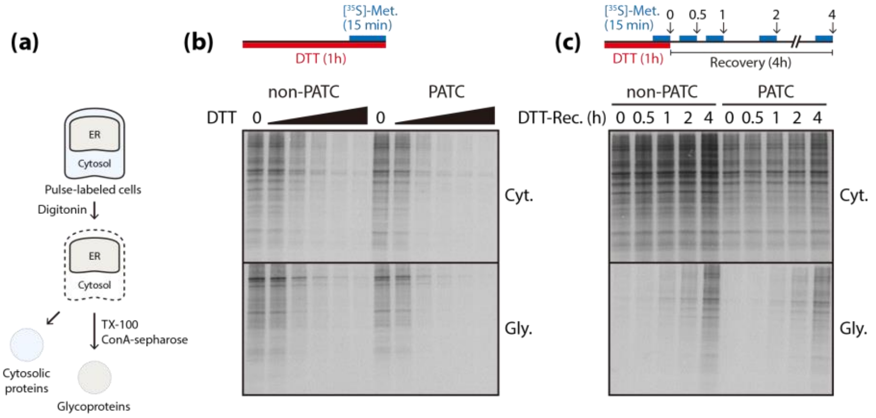

Figure S6. Effect of PATC on DTT-induced translational repression. (**a**) Experimental strategy for the selective recovery of newly synthesized cytosolic and ER proteins from semi-permeabilized cells. (**b**) Dose-dependent reduction of newly synthesized cytosolic and ER proteins was analyzed in pulse-labeled non-PATC and PATC cells treated with DTT up to 10 mM. (**c**) The restored synthesis of

cytosolic and ER protein was analyzed in non-PATC and PATC cells. Cells were treated with DTT (10 mM) for 1 h and allowed to recover for 4 h in the absence of DTT. Pulse-labeling was performed for 15 min at the indicated time points during recovery and pulse-labeled cells were analyzed as in (**a**).
